# Analysis and Risk Assessment of Total Iodine Content in Edible Seaweeds in South Korea

**DOI:** 10.3390/foods14162865

**Published:** 2025-08-19

**Authors:** YoonMi Lee, Hyung June Park, Mira Jo, Kwang Soo Ha, Jong Soo Mok

**Affiliations:** 1 Food Safety and Processing Research Division, National Institute of Fisheries Science, Busan 46083, Republic of Korea; brovojun@naver.com (H.J.P.); mirajo@korea.kr (M.J.); ksha@korea.kr (K.S.H.); 2 R&D Planning and Coordination Department, National Institute of Fisheries Science, Busan 46083, Republic of Korea; mjs0620@korea.kr

**Keywords:** inductively coupled plasma mass spectrometry, iodine, Korean diet, Korean seaweed, risk assessment

## Abstract

**Background/Objectives**: Seaweeds have recently gained global attention as sustainable and health-promoting food sources. However, seaweeds contain iodine. While iodine is a beneficial micronutrient, its excessive intake can pose health risks. Therefore, ensuring iodine safety has emerged as a critical priority. The present study aims to determine the total iodine content in five major edible seaweeds, namely laver (*Porphyra* spp.), sea mustard (*Undaria pinnatifida*), sea tangle (*Saccharina japonica*), green laver (*Enteromorpha* spp.), and hijiki (*Hizikia fusiforme*), collected from 12 coastal regions in South Korea during 2020–2024. **Methods:** A total of 348 samples were analyzed using inductively coupled plasma mass spectrometry following microwave-assisted digestion. A risk assessment was performed based on the estimated daily intake and hazard index (HI) using both the Korean Ministry of Food and Drug Safety (MFDS), EFSA (European Food Safety Authority), and the Joint FAO/WHO Expert Committee on Food Additives (JECFA) reference values. **Results:** The iodine content varied widely among the different species, with sea tangles exhibiting the highest levels (mean 2432 mg/kg dry weight). The HI values were all below 1.0, based on MFDS standards, indicating a low potential health risk. However, sea tangle exhibited values exceeding 1.0 based on the EFSA and JECFA standards. **Conclusions:** These findings highlight the need for species-specific iodine intake guidelines and safety regulations to ensure consumer protection and facilitate global seaweed trade. The present study provides a scientific basis for balancing the nutritional benefits of seaweed with the potential risks of overconsumption, contributing to the development of national dietary guidelines and providing evidence-based data for navigating international regulatory landscapes.

## 1. Introduction

Seaweeds are marine plants that grow naturally in the ocean and are rich in various functional components. Recently, seaweed plants have gained global attention as sustainable and health-promoting food ingredients. Species such as laver (*Porphyra* spp.), sea mustard (*Undaria pinnatifida*), sea tangle (*Saccharina japonica*), green laver (*Enteromorpha* spp.), and hijiki (*Hizikia fusiforme*) have long been consumed as staple food components in Asian countries, including Korea [1,2]. Seaweeds also contain abundant minerals such as calcium, magnesium, iron, zinc, potassium, and iodine, as well as vitamins A, C, E, K, and B-complex (including B1, B2, and B12). These nutrients contribute to maintaining electrolyte balance, strengthening the immune system, providing antioxidant activity, regulating blood coagulation, and supporting energy metabolism [3,4]. Notably, vitamin B12 is rarely found in plant-based foods, making seaweed a valuable source for vegetarians, such as *Porphyra* spp. [5]. Furthermore, seaweed contains both soluble and insoluble dietary fibers, which aid in improving gut health, inhibiting cholesterol absorption, and regulating blood sugar levels [6]. Alginate, abundant in brown seaweeds, is beneficial for inducing satiety and managing body weight. In addition, bioactive compounds found in seaweed, such as polyphenols, phlorotannins, and fucoidan, exhibit antioxidant, anti-inflammatory, cytoprotective, anticancer, and immunomodulatory effects [7,8,9].

Owing to the health benefits of seaweed, its consumption has expanded beyond Asia to regions such as North America, Europe, and Oceania. The European Union (EU) has recently designated seaweed as a “food of the future” [10], and the import value of edible seaweeds among the 27 EU member states increased from USD 22.88 million in 2020 to USD 33.53 million in 2022. Concurrently, seaweed cultivation and processing industries have experienced rapid growth in countries such as France, the Netherlands, and the United Kingdom [4,11]. According to the 2024 Korean fisheries export performance report, total exports of laver reached USD 997 million. Exports to the European market grew by 16.4% year-on-year, reaching USD 227 million, indicating substantial growth in Korean seaweed exports to the EU [12]. Moreover, the EU has shown growing interest in sustainable and healthy food products, including seaweed, supported by policy initiatives from the European Commission aimed at fostering the seaweed industry. The EU seaweed market is projected to reach EUR 9 billion by 2030, suggesting a favorable growth environment [13,14]. Nevertheless, seaweed consumption remains limited in Western countries, primarily owing to the lack of traditional dietary integration, sensory aversion to its unfamiliar taste and texture, insufficient public knowledge of its nutritional benefits, safety concerns, and regulatory ambiguity arising from its classification as a novel food [15,16]. Notably, this increasing interest in health-promoting diets and Asian cuisine has led to a notable increase in dietary iodine intake. To address the potential health risks associated with excessive iodine intake from seaweeds, various regulatory frameworks have been established across Western countries. For example, the EU, emphasizing informed choices, requires food products containing ≥22.5 μg iodine per 100 g dry weight (D.W.) to be labeled as “iodine-containing” and those with ≥45 μg/100 g D.W. as “iodine-rich” [10]. Germany has adopted more conservative limits, capping iodine content in food products at 20 mg/kg dDW. This regulation applies to both general foods and dietary supplements, aiming to minimize the risk of thyroid dysfunction associated with excessive iodine intake [17]. In France, the French Agency for Food, Environmental, and Occupational Health & Safety (ANSES) set the upper limit of iodine in seaweed at 2000 mg/kg, but permits up to 6000 mg/kg in brown seaweeds, which are primarily used as seasoning. This approach reflects a differentiated regulatory stance that accounts for both the frequency and quantity of consumption [18]. Australia also enforces strict regulatory standards for iodine content in imported seaweeds, restricting or prohibiting the importation of brown seaweed if its iodine content exceeds 1000 mg/kg D.W. This preventive measure aims to prevent circulating products that fail to meet domestic food safety standards and protect public health [19]. Similarly, several Western countries have established import tolerance thresholds and consumer information systems, considering both the high iodine content naturally present in seaweed and the potential health risks associated with excessive intake [16,20]. These regulatory frameworks often function as non-tariff trade barriers for seaweed-exporting countries such as South Korea [18,19]. Consequently, seaweed products intended for international markets must control iodine levels and implement rigorous analytical protocols and quality-assurance systems to comply with the specific regulatory requirements of each importing country.

Iodine is an essential micronutrient required for synthesizing thyroid hormones that regulate metabolic processes. Seaweed contains iodine at concentrations ranging from hundreds to thousands of times higher than terrestrial food, making it a valuable dietary source in regions with prevalent iodine deficiency. However, this high iodine content also poses risks, as excessive consumption may lead to adverse health outcomes. Inadequate intake can result in goiter, growth retardation, and cognitive impairment, whereas excessive intake has been associated with an increased risk of hyperthyroidism, autoimmune thyroiditis, and thyroid cancer [21]. Individuals with pre-existing thyroid conditions and older adults are particularly susceptible to iodine sensitivity, making them more vulnerable to the risks of excessive iodine exposure. Although many countries have implemented their own regulatory standards for iodine intake, there is currently no harmonized international guideline established by the Codex Alimentarius Commission (Codex). Germany has established a maximum permissible iodine content in food at 20 mg/kg D.W., whereas Australia, New Zealand, and France permit higher limits, ranging from 1000 to 2000 mg/kg D.W. According to Korean dietary surveys, the median daily iodine intake among Korean adults is estimated at 352.1 µg/day, which remains below the tolerable upper intake level (UL) set by the Ministry of Food and Drug Safety (MFDS, 2400 µg/day). This intake satisfies the World Health Organization’s (WHO) recommended nutrient intake (150 µg/day) [22,23]. Notably, approximately 77.3% of total dietary iodine intake in Korea is derived from seaweeds, highlighting the critical need for precise exposure assessments and the development of evidence-based intake guidelines.

In South Korea, annual seaweed production is approximately 1.7 million tons, representing 45% of the total volume and 10% of the total economic value of domestic seafood production. In terms of production volume, sea mustard (36%), sea tangle (31%), and laver (30%) collectively account for 97% of total seaweed yield. Regarding production value, laver contributes the largest share (71%), followed by sea mustard (12%) and sea tangle (10%). Laver is predominantly processed and distributed in seasoned or dried forms. Regionally, Jeollanam-do is the leading production area, accounting for 74% of the national output, followed by Chungcheongnam-do (10%) and Jeollabuk-do (8%) [24]. Given the increasing global demand for seaweed, ensuring iodine safety has emerged as a critical priority, particularly in anticipation of expanding Korean seaweed exports.

In this study, we analyzed the iodine content of 348 samples, representing five major seaweed species, laver, sea tangle, sea mustard, hijiki, and green laver, widely produced and consumed in South Korea, and assessed the potential health risks based on estimated dietary exposure. We collected samples to reflect key production regions and distribution channels, and our analysis accounted for species-specific and regional variations to provide accurate baseline data for risk assessment. This study offers a scientific basis for balancing the nutritional benefits of seaweed with the potential risks of excessive iodine intake. It also provides insights for supporting the development of national dietary guidelines and supplying evidence-based data for navigating international regulatory landscapes.

## 2. Materials and Methods

### 2.1. Sample Collection and Preparation

To determine the iodine content of seaweed, 348 samples were analyzed, comprising 173 farmed samples obtained from verified production sites and 175 wild-harvested specimens collected directly from 12 coastal counties and cities across South Korea during the primary seaweed harvesting season (from October 2020 to July 2024). All samples were immersed and rinsed in deionized water to remove surface impurities, then freeze-dried using a freeze-dryer (FDU-2100; EYELA, Tokyo, Japan) at temperatures below −60 °C and vacuum pressures below 10 Pa for a minimum of three days; all fresh samples were collected with a minimum weight of 20 g and then freeze-dried. After freeze-drying, the yield was approximately 10~13%. All equipment and containers used in the experimental procedures were pre-cleaned with 1% (*v*/*v*) nitric acid (suprapure 65%; Merck, Darmstadt, Germany) to minimize potential contamination.

### 2.2. Iodine Analysis

Iodine content was determined based on the Korean Food Code [25] and the Association of Official Analytical Chemists (AOAC) Official Method 2012 [26]. Freeze-dried seaweed of 0.25 g was placed into a 20 mL digestion vessel (Pyrex, New York, NY, USA), followed by the addition of 10 mL of distilled water and 2 mL of 25% (*v*/*v*) tetramethylammonium hydroxide (TMAH; Sigma-Aldrich, St. Louis, MO, USA). The mixture was digested using a microwave-assisted digestion system (UltraWAVE; Milestone, Sorisole, Italy) under the following conditions: 120 °C at 120 bar for 12 min, followed by 230 °C at 150 bar for 15 min, with a holding period of 10 min. After digestion, the sample volume was brought to 25 mL using 0.5% (*v*/*v*) TMAH. The solution was then filtered through a 0.45 μm polyvinylidene difluoride membrane filter (Merck) to remove particulates. Each sample was analyzed in triplicate using an inductively coupled plasma mass spectrometer (ICP-MS; PerkinElmer, Waltham, MA, USA). Calibration was performed using iodine standard solutions (Agilent Technologies, Santa Clara, CA, USA) at concentrations of 0.5, 1, 5, 10, 25, and 50 μg/kg. All data were processed using Easy-DOC3 software, version 3.30 (Milestone; GitHub, San Francisco, CA, USA). Final iodine concentrations are expressed on both wet-weight and dry-weight bases following moisture correction.

### 2.3. Method Validation

To validate the analytical method, the limit of detection (LOD), limit of quantification (LOQ), and recovery rate were evaluated according to AOAC guidelines [26]. The LOD and LOQ were determined from six replicate measurements of a low-concentration standard solution (1 μg/kg). Linearity was assessed using calibration curves generated from standard solutions spanning a range of concentrations, including levels relevant to the test samples. The recovery rate for iodine analysis in seaweed was calculated based on six replicate analyses of a certified reference material (CRM). The CRM employed in this study was Standard Reference Material 3232 (SRM 3232, 944 ± 88 mg/kg), provided by the National Institute of Standards and Technology (NIST).

### 2.4. Risk Assessment

To evaluate the safety of iodine intake from five types of seaweed, the estimated daily intake (EDI) of iodine was calculated using Equations (1) and (2) [27].

EDI 1 (µg/day/person) = ACDI (mg/kg D.W.) × SC (g/day)(1)

EDI 2 (µg/kg bw/day) = {ACDI (mg/kg D.W.) × SC (g/day)}/60.75 kg (person)(2)

where EDI refers to the estimated daily intake; ACDI (average concentration of detected iodine) represents the mean iodine content in seaweed (mg/kg dry weight); and SC (seaweed consumption) denotes the daily intake of seaweed (g/day) in South Korea, derived from both the average consumption by the general population and the actual intake among seaweed consumers, based on an average adult body weight of 60.75 kg. In Scenario 1, the hazard index (HI) was calculated by multiplying ACDI by SC (Equation (1)) and dividing the result by the tolerable upper intake level (UL) for iodine, which is 2400 μg/day, as established by the Korean MFDS. In Scenarios 2 and 3, HI was calculated by first multiplying ACDI by SC (Equation (2)), then dividing the result by 60.75 kg body weight, and finally comparing this value to the provisional maximum tolerable daily intake (PMTDI) of 17 μg/kg bw/day, as set by the Joint FAO/WHO Expert Committee on Food Additives (JECFA), and finally, we compared this value to the one presented in Section 3.4, Risk Assessment Result.”

### 2.5. Statistical Analysis

All seaweed samples were analyzed in triplicate, and the resulting data were statistically evaluated at a 95% confidence level using R software (version 3.6.1; http://cran.r-project.org). To assess significant differences in iodine content among seaweed groups, a one-way analysis of variance (ANOVA) was conducted using the AGRICOLAE package (CC-BY 4.0 open access, 22 October 2023, https://cran.r-project.org/web/packages/agricolae/index.html). Post hoc comparisons were performed using Duncan’s multiple range test to determine statistically significant groupings.

## 3. Results and Discussion

### 3.1. Sampling Information

**Figure 1:**
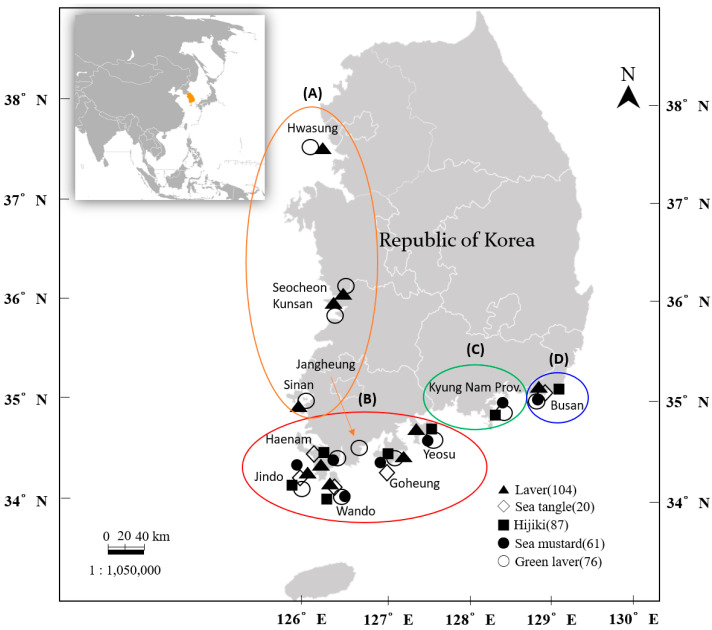
Geographic illustration of the major edible seaweed production areas in South Korea. A total of 348 seaweed samples were collected from 12 major areas.

**Figure 2:**
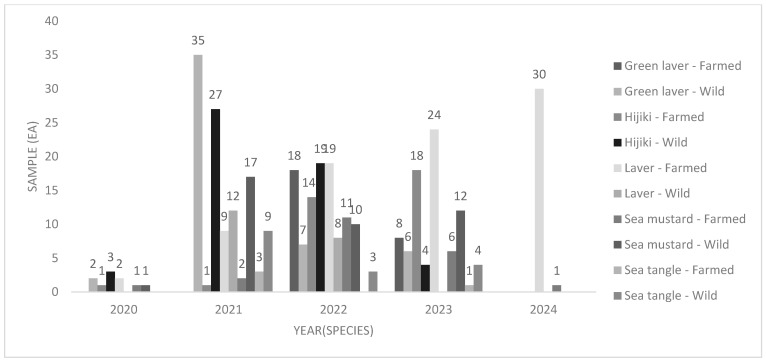
Number of samples categorized by seaweed, collection year, and origin (wild or farmed).

According to data from the Ministry of Oceans and Fisheries (MoF, 2018), we directly investigated five representative seaweed species widely produced and consumed in South Korea, harvested or purchased from 12 major coastal counties and cities between October 2020 and July 2024. The geographical distribution of the sampling sites is presented in Figure 1. The southwestern coastal region, which accounts for over 74% of the national seaweed production, includes key areas in Jeollanam-do (Haenam, Jindo, Wando, Goseong, and Yeosu), along with parts of the southeastern coast (Gyeongnam and Busan). The Western coastal sites, Hwaseong, Seocheon, Gunsan, Sinan, and Jangheung, are categorized as “others.” We collected a total of 348 seaweed samples between October 2020 and July 2024, and species and regional sample distributions are summarized in Table 1 and Figure 2.

In terms of seaweed species, the total sample distribution was as follows: laver (*n* = 104), sea tangle (*n* = 20), sea mustard (*n* = 61), hijiki (*n* = 87), and green laver (*n* = 76). As of 2017, domestic seaweed production in South Korea was dominated by sea mustard (36%), sea tangle (31%), and laver (30%). In terms of production value, laver accounted for the highest proportion (71%), followed by sea mustard (12%) and sea tangle (10%). Laver is predominantly processed into dried or seasoned products, while sea mustard is commonly produced as salted products [24].

### 3.2. Validation Method for Iodine Content of the Five Major Seaweeds

Validation results of the iodine content analysis method for the five major seaweed species are presented in Table 2. The LOQ was determined to be 2.75 μg/kg, and the LOD was 0.92 μg/kg. Calibration curves constructed using standard solutions demonstrated excellent linearity, with correlation coefficients (R^2^) averaging ≥0.9996, meeting the linearity criteria recommended by the Codex Alimentarius (R^2^ > 0.98). According to Codex method validation guidelines, acceptable recovery ranges are defined as 40–120% for concentrations of 1 μg/kg, 60–115% for 10 μg/kg, and 80–110% for 100 μg/kg. The recovery rate obtained in this study was 87.44 ± 2.74%, falling within the acceptable range and confirming the validity of the analytical method. Therefore, the iodine analysis conducted in this study satisfies international validation criteria, and the results are considered to be analytically reliable and robust.

### 3.3. Total Iodine Content of Seaweed According to Various Classification Criteria

The average iodine content of the 348 seaweed samples, classified using the cultivation method (cultivated vs. wild), collection year (2020–2024), and collection region, is summarized in Table 2. All results are expressed on a wet weight basis (mg/kg W.W.). The overall (total seaweeds) average iodine content of seaweed, categorized using the cultivation method, was 33.63 ± 60.60 mg/kg W.W., corresponding to 351.17 ± 622.99 mg/kg D.W. The detection range was 0.401–374.866 mg/kg W.W. (8.154–4164.765 mg/kg D.W.). We analyzed the iodine concentrations in the five major seaweed species, and the results are summarized in Table 3. We reported these measurements based on both wet weight (W.W) and dry weight (D.W) to account for moisture variability.

Among the species analyzed, Laver (*Porphyra* spp.) exhibited iodine concentrations ranging from 0.40 to 8.15 mg/kg W.W and 23.45 to 412.19 mg/kg D.W, with mean values of 6.14 ± 5.09 mg/kg and 100.18 ± 78.60 mg/kg, respectively. Similarly, sea tangle (*S. japonica*) contained considerably elevated iodine levels, with values of 24.45–284.34 mg/kg W.W. and 374.87–4164.76 mg/kg D.W. The mean iodine content was 227.92 ± 97.50 mg/kg W.W and 2432.11 ± 1110.25 mg/kg D.W. Therefore, sea tangle exhibited the highest iodine concentrations among the tested species. Other species, including sea mustard (*U. pinnatifida*), exhibit iodine levels of 3.29–39.25 mg/kg W.W and 39.06–455.62 mg/kg D.W., with mean values of 12.82 ± 7.42 mg/kg and 176.89 ± 88.91 mg/kg, respectively. For Hijiki (*H. fusiforme*), the iodine concentrations were 8.74–143.72 mg/kg W.W and 159.23–1547.71 mg/kg D.W, with mean values of 60.49 ± 35.08 and 526.57 ± 282.16 mg/kg, correspondingly. Green laver (Enteromorpha spp.) displayed the lowest iodine levels among the tested species, ranging from 0.72 to 12.75 mg/kg W.W and 19.49 to 366.25 mg/kg D.W, with mean values of 6.05 ± 4.10 and 86.11 ± 59.85 mg/kg, respectively.

#### 3.3.1. Variation in Iodine Content According to the Cultivation Method (Wild and Farmed)

We analyzed 348 total seaweed samples, including 175 wild-harvested (20 laver, 12 sea tangle, 40 sea mustard, 53 hijiki, and 50 green laver) and 173 farmed samples, from verified producers (84 laver, 8 sea tangle, 21 sea mustard, 34 hijiki, and 26 green laver). Laver was the most extensively cultivated species among the analyzed samples. Details of the collected seaweed are shown in Table 1 and Figure 2. The iodine content of wild seaweed ranged from 0.590 to 355 mg/kg W.W., averaging 41.3 mg/kg W.W., while that of farmed seaweed ranged from 0.401 to 375 mg/kg W.W., averaging 25.5 mg/kg W.W. Overall, farmed seaweed exhibited approximately 61% higher iodine content than wild seaweed. When analyzed by species, wild and farmed iodine concentrations were 219 vs. 264, 58.1 vs. 64.2, 11.0 vs. 15.9, 5.54 vs. 7.04, and 3.92 vs. 6.67 mg/kg W.W. for sea tangle, hijiki, sea mustard, green laver, and laver, respectively. Farmed seaweed exhibited iodine concentrations that were 10–70% higher than those of their wild counterparts, with the smallest differences observed in sea tangle and hijiki (10–20%) and the largest in laver (approximately 70%). Among all species, sea tangle consistently exhibited the highest iodine levels, with more than 45 times the iodine content of laver. These findings indicate that the significantly elevated iodine levels in sea tangle are the primary factor influencing the overall results of this study.

Numerous studies have reported significant differences in iodine content between wild and farmed seaweed. Schiener et al. [28] reported, that in aquaculture settings for sea mustard, factors such as elevated nutrient inputs (particularly nitrogen and phosphorus) from surrounding waters, controlled water temperatures, and increased cultivation density can contribute to the accumulation of iodine and other trace elements. Specifically, aquaculture environments, which are characterized by shallow water depth and limited water circulation, may promote continuous iodine availability, thereby facilitating its accumulation in seaweed tissues. Roleda et al. [29] investigated the iodine content of both brown and red algae widely produced in the North Atlantic, distinguishing between wild and farmed specimens. Their findings indicate that wild species exhibit lower iodine concentrations than farmed ones, which are consistent with our results. However, wild seaweed is continuously subjected to environmental stress such as tidal fluctuations, intense solar radiation, and periodic dehydration. Seaweed accumulates high levels of iodine as part of its antioxidant defense mechanism, utilizing iodine to neutralize reactive oxygen species (ROS) generated under these stress conditions [30]. This mechanism is particularly prominent in brown algae, where iodine is believed to function through a vanadium-dependent haloperoxidase system, facilitating ROS scavenging. This may explain the discrepancies between our findings and previous studies. These findings suggest that the iodine content of wild and farmed seaweed differs according to species, cultivation conditions, nutritional factors, and geographic region. Meanwhile, various studies have been conducted comparing wild and farmed seaweed populations. Graf et al. [31] performed a comparative genomic analysis of wild, farmed, and introduced populations of sea mustard (*Undaria pinnatifida*) and found that farmed populations exhibited unexpectedly high genetic diversity. This was attributed to the mixing of parental individuals from diverse sources during the seeding process. The iodine content of seaweed varies substantially depending on the growth and harvest periods, which are strongly influenced by seasonal marine environmental factors such as water temperature, light intensity, nutrient concentration, and growth cycle dynamics [32]. In this study, we monitored monthly iodine concentrations from October 2021 to July 2024, with the following number of samples collected per month: October (*n* = 21), November (*n* = 38), December (*n* = 61), January (*n* = 66), February (*n* = 57), March (*n* = 52), April (*n* = 36), May (*n* = 7), June (*n* = 5), and July (*n* = 5). The five tested seaweed species displayed distinct seasonal growth and harvesting patterns that correspond to marine environmental conditions such as seawater temperature, solar radiation, and tidal currents. Specifically, species such as laver and green laver favor colder seasons, exhibiting rapid growth during the winter months. For these species, growth and harvest periods often overlap, typically occurring between November and April. Moreover, sea mustard, sea tangle, and hijiki are predominantly winter-growing species, with hijiki typically harvested in February and March, sea mustard from March to May, and sea tangle from March to June. Our results reveal that sea tangle exhibits an average iodine concentration of 234.15 and 278.90 mg/kg in November and December, respectively, corresponding to the early growth phase. This was followed by a decrease to 183.98 mg/kg during the harvesting season, representing a reduction of over 30%. In contrast, hijiki exhibited lower iodine levels during the early growth period (45.16–42.99 mg/kg in December–January), with significantly higher concentrations during the harvest season (68.20–80.97 mg/kg in February–March), suggesting increased iodine accumulation toward the latter growth stages. These findings suggest that differences in iodine content between wild and farmed seaweed may be partially explained by growth stage and harvest timing. This highlights the influence of seasonal and physiological factors on iodine accumulation.

Rehder [28] reported that seaweed, particularly brown algae, utilizes iodine as an antioxidant to scavenge ROS under environmental stress conditions such as low seawater temperatures and limited light availability, especially during winter and early spring. The activation of this antioxidant defense mechanism contributes to elevated iodine accumulation in algal tissues during these periods. However, as seawater temperature and light intensity increase with the onset of spring, seaweed growth rate accelerates. During this phase, metabolic activity shifts toward organic compound synthesis required for rapid cell division and tissue expansion, leading to a relative decrease in iodine accumulation [30]. In addition, the tissue dilution effect caused by rapid growth can reduce the iodine concentration per unit D.W. In particular, high-temperature and high-light-intensity environments increase seaweed metabolic rate, which promotes iodine consumption and further reduces iodine concentration in the tissue. Furthermore, iodine content tends to increase again in autumn. Owing to these seasonal fluctuations, seaweed’s nutritional value can vary depending on harvest timing and environmental conditions. Therefore, when considering seaweed as a source of iodine, harvest timing is an important factor to consider.

#### 3.3.2. Temporal Variation in Iodine Content According to the Collection Year (2021–2024)

The average iodine content of seaweed by harvest year was 65.6 mg/kg W.W. in 2021, 65.3 mg/kg W.W. in 2022, 47.0 mg/kg W.W. in 2023, and 67.2 mg/kg W.W. in 2024, with the lowest concentration observed in 2023. Excluding 2023, iodine levels remained relatively consistent throughout the other years. When analyzed by species, the average iodine content of sea tangle was 235, 254, 140, and 229 mg/kg W.W. in 2021, 2022, 2023, and 2024, respectively (mean: 214.5 mg/kg W.W.). For hijiki, the values were 72.2, 51.3, and 67.1 mg/kg W.W. in 2022, 2023, and 2024, respectively (mean: 63.5 mg/kg W.W.). Sea mustard exhibited concentrations of 10.27, 10.8, 16.2, and 12.4 mg/kg W.W. across 2021–2024, respectively (mean: 12.5 mg/kg W.W.). Green laver exhibited 5.41, 5.57, 6.5, and 19.5 mg/kg W.W. across 2021–2024, respectively, with a relatively high value observed in 2024 (mean: 9.3 mg/kg W.W.). Laver had values of 4.48, 4.85, 5.41, and 7.95 mg/kg W.W. during the same period (mean: 5.7 mg/kg W.W.). Notably, both green laver and laver, species with comparatively low baseline iodine concentrations, exhibited a substantial increase in 2024. However, Roleda et al. [31] reported that the brown algae Saccharina spp. (sea tangle) generally contains higher iodine concentrations than red algae such as laver. While significant variation may exist depending on geographic harvest location, we did not observe clear differences by season or year of collection in the present study.

#### 3.3.3. Geographical Variation in Iodine Content According to the Collection Area

Excluding Gyeongnam, where samples of laver or sea tangle were not collected, sea mustard, green laver, and hijiki exhibited the highest iodine concentrations in Busan. In contrast, sea tangle and laver exhibited the highest levels in Jindo, Jeollanam-do. For sea mustard, the iodine content in Busan was 17.8 mg/kg W.W., compared to 10.6 mg/kg W.W. in Wando, Jeollanam-do, the region with the lowest concentration, indicating minimal regional variation. However, we observed more pronounced differences within Jeollanam-do itself. For example, the iodine content in hijiki collected from Haenam was approximately 1.5 times higher than that from other areas. Moreover, sea tangle from Jindo exhibited a value of 335 mg/kg W.W., which was almost 25 times higher than the 83 mg/kg W.W. recorded in Goheung. Laver showed considerable regional variability, with iodine concentrations of 1.81–7.21 mg/kg W.W. (mean: 5.04 mg/kg W.W.). Notably, within Jeollanam-do, laver collected from Jindo contained 7.21 mg/kg W.W., more than four times the concentration presented in samples from Yeosu (1.81 mg/kg W.W.). According to a recent study on the average iodine content of laver produced or harvested in different countries, the iodine concentrations in New Zealand, Ireland, Italy, and Spain are 45.0–64.0, 56.0, 12.02, and 35.0–102.0 mg/kg D.W., respectively [33]. In addition, a report by the Korean MFDS for 2006–2012 indicated the average iodine content of domestically produced laver to be 51.6 mg/kg D.W., with a broad range of 12.8–174 mg/kg D.W., depending on the production period and region. Iodine content is also influenced by cultivation depth. For example, sugar kelp (*Saccharina latissima*) seaweed cultivated at a depth of 9 m contains significantly higher iodine concentrations than that planted at 1 m, confirming that water depth plays a role in iodine accumulation [34].

Sea tangle contains an average iodine concentration of 7.8 mg/g or higher based on D.W., with some varieties reported to contain up to 12 mg/g [35]. This high accumulation capacity is attributed to the ability of sea tangle to efficiently absorb iodine from seawater and convert inorganic iodine into organic iodine compounds for storage. This process is mediated by haloperoxidase enzymes located in the cell wall [36]. The iodine stored in the cell wall functions as an antioxidant, mitigating physiological stress caused by external environmental factors. Laver has a limited capacity for iodine uptake and accumulation [37]. This is primarily attributed to its weak or inactive haloperoxidase system and its typical habitat in shallow coastal waters, where iodine concentrations are relatively low. As a result, the average iodine content of laver ranges from 16 to 100 μg/g D.W. In comparison, green laver, a representative species of green algae, contains iodine levels of 50–200 μg/g D.W. According to a study by Milinovic et al. [38], the iodine content of green algae, *Ulva rigida*, is only 33 μg/g D.W., whereas that of brown algae, *Bifurcaria bifurcata* and *Fucus vesiculosus*, was 391 and 352 μg/g D.W., respectively. The authors concluded that the elevated iodine content in brown algae is influenced by a combination of environmental factors, including species-specific characteristics, growth duration, seawater temperature, salinity, and harvesting location. Consistent with these findings, we also observed substantial variation in both average and maximum iodine content based on harvest location (e.g., Gochang, Wando, and Jindo in Jeollanam-do) and harvest period (from January to April). Notably, sea tangle and hijiki exhibited fluctuations of more than tenfold depending on the time of harvest. Given this high variability, accurate safety assessments and exposure estimations are essential when evaluating seaweed as a dietary source of iodine.

In conclusion, brown algae, such as sea tangle, exhibit optimized physiological mechanisms for iodine accumulation and inhabit ecological niches conducive to iodine uptake, resulting in markedly high iodine concentrations. In contrast, species such as sea mustard, laver, and green laver possess relatively low iodine levels, primarily owing to differences in enzymatic systems and marine environmental conditions. These interspecies differences in iodine content reflect a complex interplay of evolutionary divergence, cell wall structure, biochemical traits, and ecological adaptation. Accordingly, developing species-specific consumption guidelines that account for these variations in iodine content are essential to ensure safe and nutritionally appropriate seaweed intake.

### 3.4. Risk Assessment Result

The average requirement (AR) and the tolerable UL serve as key benchmarks in assessing the adequacy and safety of nutrient intake. These reference values are widely applied in frameworks such as Dietary Reference Intake (DRI) developed by the Institute of Medicine (IOM) under the US National Academies, Dietary Reference Values (DRVs) established by the European Food Safety Authority (EFSA) [39], and the international standards provided by the Joint FAO/WHO Expert Committee on Food Additives (JECFA), which evaluates the safety of food additives and contaminants. In this study, based on the iodine content of five Korean seaweed species, we conducted a risk assessment by referencing the ULs established in the DRI, EFSA DRVs, and JECFA standards [40,41]. Table 4 presents the results of the dietary risk assessment for seaweed consumption. The average iodine content by species was 100.2, 2432.1, 176.9, 526.6, and 86.1 mg/kg D.W. for laver, sea tangle, sea mustard, hijiki, and green laver, respectively. The average daily intake of each seaweed species by the general Korean population was 1.02, 0.67, 0.72, 0.09, and 0.38 g/day/person for laver, sea tangle, sea mustard, hijiki, and green laver, respectively. Among actual consumers (those who reported consuming the corresponding seaweed, the average intake was significantly high, with values of 2.94, 15.79, 4.83, 18.3, and 24.39 g/day/person/D.W. for laver, sea tangle, sea mustard, hijiki, and green laver, respectively. The EDI values (µg/kg body weight/day) for the general population and actual consumers were as follows: laver: 1.69 (4.85); sea tangle: 26.88 (632.01); sea mustard: 2.09 (14.06); hijiki: 0.78 (158.63); green laver: 0.54 (34.57).

The total EDIs of iodine in South Korea were higher than the AR established in the IOM (95 µg/day/adults) and the recommended daily intake recommended by the WHO (150 μg/day/adults). In Scenario 1, based on the Korean MFDS-UL (2400 µg/day/person), the HIs (average) for all seaweed species remained below 1. However, among the consumer group, the HI values for hijiki (4.02) and sea tangle (16.00) exceeded 1. In Scenario 2, based on the EFSA-UL (600 µg/day/person), the HI values for individual seaweed species in the general population range were 0.05–2.72, with a cumulative HI of 3.24, remaining over the safety threshold. In the consumer group, the HIs for individual items were 0.49–63.99, with a total cumulative HI of 85.47, significantly exceeding the recommended limit. Notably, sea tangle exhibited the highest HI range (0.68–63.99), likely owing to both its elevated iodine content and higher consumption levels. When evaluated against the standards set by the Korean MFDS, the HI for each seaweed and total intake remained below 1, suggesting a low risk of adverse effects due to excessive iodine intake. However, when applying the EFSA-UL, the HI for sea tangle solely was 2.72, and the cumulative HI for all seaweed species reached 3.24. These results indicate a potential health risk associated with excessive iodine intake, particularly from high-iodine species such as sea tangle and in cases of combined seaweed consumption.

In Scenario 3, based on the total population, the HI for all seaweed species except sea tangle remained below 1. However, among the consumer group, the HI values for hijiki, green laver, and sea tangle exceeded 1. Notably, sea tangle exhibited the highest HI range, 0.68–37.18, which can be attributed to both its elevated iodine content and higher consumption levels. According to the WHO, the recommended daily intake of iodine for adults is 150 μg/day, and the UL is 1100 μg/day. When evaluated against the standards set by the Korean MFDS, the HI for each seaweed and total intake remained below 1, suggesting a low risk of adverse effects due to excessive iodine intake. However, when applying the JECFA PMTDI, the HI for sea tangle solely was 1.58, and the cumulative HI for all seaweed species reached 1.88. These results indicate a potential health risk associated with iodine intake, especially from high-iodine species such as sea tangle, and when multiple types of seaweed are consumed together. Although the risk assessment based on the Korean MFDS standard (2400 µg/day) suggests a relatively low risk, a more conservative approach is recommended when compared to European and international standards (Table 4).

Aakre et al. [42] conducted a randomized crossover study involving 20 healthy Danish women (aged 24–30) and reported that the bioavailability of iodine (total dose: 231 μg) was approximately 75% following the consumption of sea mustard salad and sushi. Although this bioavailability was lower than the 97% bioavailability observed with potassium iodide (KI) supplements containing the same iodine dose, it was still considered a substantial absorption rate. Furthermore, González et al. [43] evaluated iodine content and potential human exposure risks associated with the consumption of sea mustard and sea tangle, owing to increased dietary popularity. They analyzed a total of 30 seaweed samples sourced from various regions, including Asia and Europe, using the oxidation–reduction titration method. European sea tangle had the highest iodine content among all samples, averaging 27.7 ± 5.4 mg/kg D.W. European sea mustard also exhibited higher iodine concentrations than the Asian counterparts. Notably, sea mustard demonstrated significant differences in iodine content depending on the origin (*p* < 0.05). In addition, daily consumption of 4 g of European sea tangle provides approximately 111 μg of iodine, which approaches the recommended daily intake for adults of 150 μg/day. The WHO has established an UL of 1100 μg/day for adults, highlighting that while seaweed can serve as a valuable dietary source of iodine, excessive intake may pose health risks. Accordingly, routine monitoring of iodine content in seaweed products is essential. Particular attention should be given to the consumption of high-iodine species such as sea tangle, hijiki, and sea mustard, which should be consumed in moderation to avoid excessive iodine exposure.

In a large-scale cohort study involving 190,524 individuals in Korea, Park et al. [44] investigated the relationship between iodine intake and thyroid disease. They demonstrated that excessive iodine intake was not significantly associated with an increased risk of thyroid dysfunction. This finding may be attributed to the traditional dietary patterns and adaptive iodine metabolism in the Korean population. These results highlight the importance of developing population-specific dietary guidelines and implementing policy measures such as warning labels for seaweed products with high iodine content. However, daily iodine intake varies substantially by country, region, and dietary habits. In Korea, the average daily iodine intake is estimated to be 400–500 μg, with seaweed accounting for approximately 77% of total dietary iodine intake. Among seaweed sources, sea mustard, sea tangle, and laver contribute 44.0%, 20.4%, and 13.1% of the total iodine intake, respectively [23]. In contrast, in countries such as the United States, Canada, and several European nations, dairy products, including milk and cheese, are the primary contributors to dietary iodine. Nevertheless, the recent WHO/Europe and Iodine Global Network (IGN) report confirms that mild iodine deficiency remains a widespread concern in the European region, despite some improvements over the years [45]. In addition to iodine, South Korea has set a standard of 0.3 mg/kg for cadmium, a harmful substance found in laver, while the EU and China have expanded the standard to include seaweed varieties.

Our results suggest that future research should investigate changes in bioavailability depending on cooking methods (e.g., boiling and blanching), as not all iodine in seaweed is absorbed. Re-evaluating seaweed consumption safety, considering the iodine retention rate after cooking and human absorption rates, and conducting a survey on actual consumption patterns for each type of seaweed are necessary. Moreover, customized risk assessments targeting high-risk groups, such as individuals with thyroid disorders and pregnant women, are also necessary. Given the evidence linking seaweed consumption and thyroid function (changes in blood TSH, T3, and T4 levels), there is a need to address inconsistent international standards. This should involve conducting comparative studies on iodine standards between countries and providing policy recommendations for establishing seaweed consumption guidelines. In the present study, we quantitatively evaluated and analyzed the iodine content of major seaweeds in Korea and the potential health risks associated with consumption. Our results reveal a low risk based on the Korean MFDS standards. However, according to JECFA standards, we identified a risk of excessive consumption, particularly associated with sea tangle. Furthermore, considering the expanding export markets, ensuring scientific safety is essential to build international trust.

## 4. Conclusions

Seaweed, a traditional Korean food ingredient, is gaining global recognition alongside other iconic Korean foods such as kimchi and fermented soybean paste. Brown seaweed species, such as sea mustard, sea tangle, and hijiki, have strong potential as functional food ingredients, owing to their rich iodine content, and are gaining international attention as dietary iodine sources. However, given the high variability in iodine accumulation, depending on growing conditions, a scientific understanding of the underlying environmental influences, including light intensity, nutrient availability, temperature, and water depth, is essential for ensuring product safety and consistency [30]. In the present study, we analyzed the iodine content of 348 samples, representing five edible seaweed species (laver, sea tangle, sea mustard, hijiki, and green laver), collected from the major coastal regions of South Korea over four years (2021–2024), and then conducted a health risk assessment based on the results. Among these species, sea tangle exhibited the highest average iodine concentration, followed by hijiki, sea mustard, laver, and green laver. Brown and red algae generally demonstrated more iodine accumulation than green algae, with notable variations attributed to environmental factors such as seawater temperature, salinity, and depth, cultivation method, and harvesting area and period. These interspecies and environmental differences are critical for accurate iodine exposure estimation and risk assessment related to seaweed consumption.

We also conducted a risk assessment based on both the Korean MFDS, EFSA, and JECFA standards. Sea tangle exhibited significantly higher iodine content and consumption levels than other seaweeds, resulting in the highest HI across all evaluation criteria. While the Korean MFDS guideline (UL of 2400 µg/day) suggests that the HI values for individual seaweed species, as well their cumulative intake, generally remain below or near the safety threshold of 1, suggesting a low health risk for the general population, more stringent international standards, such as those from EFSA (UL of 600 µg/day) and JECFA, reveal considerably elevated HI values, especially for sea tangle. These elevated values often exceed the safety limit, indicating a potential risk of iodine overconsumption, particularly in consumer subgroups that ingest multiple types or higher amounts of seaweeds. Therefore, although typical consumption may be safe under domestic guidelines, adopting a more conservative approach to seaweed intake, especially limiting high-iodine species like sea tangle, is advised. Such an approach would minimize the potential health risks associated with excessive iodine intake.

The EDI by species increased progressively across species, from laver to sea mustard, hijiki, green laver, and ultimately sea tangle, which exhibited the highest value. All species, except sea tangle, fell within the tolerable intake range of JECFA. However, sea tangle was estimated at 1632.61 μg/day, approximately 70% of the WHO upper intake limit (1100 μg/day), warranting caution. These findings align with data from the Korea National Health and Nutrition Examination Survey (2016–2022), which reported a mean iodine intake of 559.16 ± 13.15 μg/day, exceeding the recommended intake (150 μg/day). However, only 37.6% of the population reported consuming seaweed, indicating that average intake may be inflated by high-consuming subgroups. Notably, sea tangle is commonly used in soups, and cooking processes such as boiling and blanching can reduce iodine content by 30–90% [46], suggesting that bioavailability-adjusted risk assessments are needed. Milinovic et al. [38] similarly emphasized the impact of processing methods on iodine variability, highlighting the importance of accounting for pre- and post-processing changes in exposure evaluations. Therefore, future research should focus on refining dietary risk models by incorporating processing effects and bioavailability data and aligning iodine measurements with actual consumption patterns.

Our study’s findings provide a foundation for developing standardized cultivation protocols, iodine content prediction models, and international certification systems for quality control. These efforts are essential for establishing a stable global supply chain for K-Food and for promoting a safe, functional, and reliable image of Korean seaweed to consumers worldwide.

## Figures and Tables

**Table 1:** Five major seaweed collections based on the regions in South Korea.

	KN	BS	JN					Others	Total
			GH	YS	WD	JD	HN		
Laver	0	4	9	1	10	35	26	19	104
Sea tangle	0	5	3	0	7	2	3	0	20
Sea mustard	2	8	15	2	22	4	8	0	61
Hijiki	1	3	21	6	35	18	3	0	87
Green laver	2	3	8	2	30	13	15	3	76
Total	5	23	56	11	104	72	55	22	348

KN: Kyungnam Province; BS: Busan; JN: Jeonnam Province (GH: Goheung; YS: Yeosu; WD: Wando; JD: Jindo; HN: Haenam). Others (HS: Hwasung; SC: Seocheon; KS: Kunsan; SA: Sinan; JH: Jangheung). In total, two hundred and ninety-eight samples from JN, twenty-three samples from BS, five from KN, and twenty-two from others (four from HS, nine from SC, five from KS, two from SA, and two from JH).

**Table 2:** Quality parameters of analytical methods for iodine determination.

**CRM** **(SRM 3232, ** 944 ± 88 mg/kg**)**	**LOD ^a^**	**LOQ ^b^**	**Linearity**	**Recovery**
	**(µg/kg)**	**(µg/kg)**	**(R^2^)**	**(%)**
	0.92	2.75	0.9996	87.44 ± 2.74

CRM: Certified reference materials (standard reference material (SRM) 3232). ^a^ LOD: Limit of detection = 3.143 × σ. ^b^ LOQ: Limit of quantification = 10 × σ. (σ: Standard deviation of seven replicate measurements of the standard solution).

**Table 3:** Summary of iodine concentrations (mg/kg) of five major seaweeds based on growth methods, harvest years, and sampling regions.

Species	Growth Method				Harvest Year								Sampling Region															
	Wild		Farmed		2020–21		2022		2023		2024		KN		BS		JN										Others	
																	GH		YS		WD		JD		HN			
	W.W	D.W	W.W	D.W	W.W	D.W	W.W	D.W	W.W	D.W	W.W	D.W	W.W	D.W	W.W	D.W	W.W	D.W	W.W	D.W	W.W	D.W	W.W	D.W	W.W	D.W	W.W	D.W
Laver	3.92	69.6	6.67	107	4.48	78	4.85	71.5	5.41	90.1	7.95	130	- ^a^	-	3.96	43.94	5.88	97.02	1.81	32.59	4.87	89.60	7.21	102.32	6.7	110.78	4.85	104.18
Sea tangle	219	2308	264	2929	235	2540	254	2079	140	1888	229	3329	-	-	268	3102	83	1195	-	-	241	2531	318	2023	215	2594	-	-
Sea mustard	11	157	15.9	205	10.72	143	10.8	177	16.2	185	12.4	196	16.7	235	17.8	201.1	12.1	185	12.9	170.8	10.6	144.1	14.5	207.5	13.5	199.3	-	-
Hijiki	58.1	505	64.2	561	72.2	620	51.3	438	67.1	599	-	-	77.3	557	98.4	770	52.5	461	50.5	557	67.2	558	49.5	453	80.0	752	-	-
Green laver	5.54	70.3	7.04	116	5.41	60.2	5.57	76.3	6.5	104	19.5	366	6.23	132	9.84	165	5.75	74	9.43	130	6.58	86	4.98	70	5.06	74	3.92	100

^a^-: No sampling from these regions. KN: Kyungnam Province, BS: Busan, JN: Jeonnam Province (GH: Goheung; YS: Yeosu; WD: Wando; JD: Jindo; HN: Haenam). Others (HS: Hwasung; SC: Seocheon; KS: Kunsan; SA: Sinan; JH: Jangheung); D.W.: Dry weight; W.W.: Wet weight.

**Table 4:** Risk assessment of iodine intake for the five major seaweeds. Hazard index evaluation of two scenarios using MFDS, EFSA, and JECFA reference values.

Species	ACDI (mg/kg dw)	SC (g/day) ^a^	EDI 1 (µg/day/person) ^c^		EDI 2 (µg/kg bw/day) ^d^		Scenario 1 ^e^		Scenario 2 ^f^		Scenario 3 ^g^	
							HI (Korean MFDS)		HI (EFSA)		HI (PMTDI)	
		Avg. ^b^	Avg.	Act.	Avg.	Act.	Avg.	Act.	Avg.	Act.	Avg.	Act.
Laver	100.2	1.02	102.44	294.69	1.69	4.85	0.04	0.12	0.17	0.49	0.10	0.29
Sea tangle	2432.1	0.67	1632.61	38,393.31	26.88	632.01	0.68	16.00	2.72	63.99	1.58	37.18
Sea mustard	176.9	0.72	127.12	854.19	2.09	14.06	0.05	0.36	0.21	1.42	0.12	0.83
Hijiki	526.6	0.09	47.50	9636.62	0.78	158.63	0.02	4.02	0.08	16.06	0.05	9.33
Green laver	86.1	0.38	32.86	2100.32	0.54	34.57	0.01	0.88	0.05	0.88	0.03	2.03
Total							0.81	21.37	3.24	85.47	1.88	49.31

^a^ Seaweed consumption was based on the “Korea National Health and Nutrition Examination Survey (KNHANES) 2023.” ^b^ Avg.: Average daily intake of seaweed by the Korean population. ^c^ EDI 1 was calculated by {ACDI X SC of Avg. or Act}. ^d^ EDI 2 was calculated by {ACDI X SC of Avg. or Act.}/average Korean adult body weight (60.75 kg). ^e^ Scenario 1 is the result of HI = EDI/2400 µg/day/person. ^f^ Scenario 2 is the result of HI = EDI/600 µg/day/person. ^g^ Scenario 3 is the result of HI = EDI 2/provisional maximum tolerable daily intake of iodine (PMTDI) from JECFA (17 µg/kg bw/day). Hi, Hazard Index; EFSA, European Food Safety Authority; JECFA, Joint FAO/WHO Expert Committee on Food Additives; ACDI, average concentration of detected iodine; SC, seaweed consumption; ED, estimated daily intake; IOM, Institute of Medicine.

## Data Availability

The original contributions presented in this study are included in the article/supplementary material. Further inquiries can be directed to the corresponding author.
